# Playing around the anaerobic threshold during COVID-19 pandemic: advantages and disadvantages of adding bouts of anaerobic work to aerobic activity in physical treatment of individuals with obesity

**DOI:** 10.1007/s00592-021-01747-1

**Published:** 2021-05-28

**Authors:** Alberto Salvadori, Paolo Fanari, Paolo Marzullo, Franco Codecasa, Ilaria Tovaglieri, Mauro Cornacchia, Ileana Terruzzi, Anna Ferrulli, Patrizia Palmulli, Amelia Brunani, Stefano Lanzi, Livio Luzi

**Affiliations:** 1 Department of Pulmonary Rehabilitation, Istituto Auxologico Italiano IRCCS, Verbania (VB), Italy; 2 Division of General Medicine, Ospedale S. Giuseppe, Istituto Auxologico Italiano, via Cadorna 90, 28824 Piancavallo Di Oggebbio (VB), Italy; 3 Department of Translational Medicine, University of Piemonte Orientale, via Solaroli 17, 28100 Novara, Italy; 4 Department of Rehabilitation Medicine, Istituto Auxologico Italiano IRCCS, Verbania (VB), Italy; 5 Division of Angiology, Heart and Vessel Department, Lausanne University Hospital, Lausanne, Switzerland; 6 Department of Endocrinology, Nutrition and Metabolic Diseases, IRCCS MultiMedica, Via Milanese 300, Sesto San Giovanni, Italy; 7 Department of Biomedical Sciences for Health, University of Milan, Milan, Italy

**Keywords:** Anaerobic threshold, High-intensity, Obesity, Physical exercise, Rehabilitation, covid-19

## Abstract

**Introduction:**

Obesity is a condition that generally limits work capacity and predisposes to a number of comorbidities and related diseases, the last being COVID-19 and its complications and sequelae. Physical exercise, together with diet, is a milestone in its management and rehabilitation, although there is still a debate on intensity and duration of training. Anaerobic threshold (AT) is a broad term often used either as ventilatory threshold or as lactate threshold, respectively, detected by respiratory ventilation and/or respiratory gases (VCO_2_ and VO_2_), and by blood lactic acid.

**Aims and methodology:**

This review outlines the role of AT and of the different variations of growth hormone and catecholamine, in subjects with obesity vs normal weight individuals below and beyond AT, during a progressive increase in exercise training. We present a re-evaluation of the effects of physical activity on body mass and metabolism of individuals with obesity in light of potential benefits and pitfalls during COVID-19 pandemic. Comparison of a training program at moderate-intensity exercise (< AT) with training performed at moderate intensity (< AT) plus a final bout of high-intensity (> AT) exercise at the end of the aerobic session will be discussed.

**Results:**

Based on our data and considerations, a tailored strategy for individuals with obesity concerning the most appropriate intensity of training in the context of rehabilitation is proposed, with special regard to potential benefits of work program above AT.

**Conclusion:**

Adding bouts of exercise above AT may improve lactic acid and H^+^ disposal and improve growth hormone. Long-term aerobic exercise may improve leptin reduction. In this way, the propensity of subjects with obesity to encounter a serious prognosis of COVID-19 may be counteracted and the systemic and cardiorespiratory sequelae that may ensue after COVID-19, can be overcome. Individuals with serious comorbidities associated with obesity should avoid excessive exercise intensity.

## 1. Introduction

### Obesity, exercise, and COVID-19

In humans, both physiological and neuroendocrine factors act to control the amount of biological activity of white adipose tissue. In subjects with obesity, a reduced metabolic response to sympathetic nervous system (SNS) activity is known [1, 2] , with a blunted lipolytic action on visceral adiposity [3, 4]. Several hormones including corticosteroids, growth hormone (GH), androgens and estrogens are less responsive to exercise in subjects with obesity [5, 6]. All the above contribute to regulating lipid metabolism in response to exercise training. Yet, regular exercise is an important step, together with diet, in the management and rehabilitation strategies of individuals with obesity, due to its ability to modulate both components of the energy balance equation, namely energy intake and energy output [6]. Furthermore, we recently reported on the positive immunomodulatory effects of physical exercise protecting against COVID-19 [7]. Since obesity is at the same time a risk factor for the contagion by SARS-CoV_2_ and predisposes infected individuals to a worse prognosis [8], herein we present several considerations on the binomial obesity-exercise intensity and COVID-19.

In general, subjects with obesity have a decreased work capacity when compared to lean subjects, mostly due to an increased cardiac left ventricular mass and wall thickness with abnormalities in diastolic filling [9]. Moreover, they must overcome a decreased compliance in respiratory system [10]. Maximal sustainable work outputs in young subjects with obesity are similar to those of lean age-matched controls, but they attain the anaerobic threshold (AT) at significant lower outputs [11]. This means that endurance time at maximal effort is shorter, and therefore, that work capacity is lower [12]. Older subjects with obesity show a significant reduction in maximal sustainable work capacity and reach earlier AT, reflecting a more severe reduction in physical performance as compared to controls [13].

Furthermore, individuals with obesity have an altered dynamic of pulmonary ventilation, which is a potential cause of the worse clinical picture of COVID-19 [8]. During a progressive exercise, the delta increment of oxygen consumption is the same in subjects with obesity and in normal weight subjects. Nevertheless, obesity implies a constant significant greater absolute amount of oxygen at each correspondent external work output. This suggests that gross mechanical efficiency (Watts/VO_2_) is lower in individuals with obesity despite similarities in net mechanical efficiency (Watts/VO_2_—VO_2_ freewheeling) when compared to controls [11]. The underlying mechanism involves a higher peripheral oxygen uptake in individuals with obesity than in controls. This is partly due to a different pattern in circulating endothelin-1/NO concentrations, with a decrease in the former and a higher level of the latter in subjects with obesity [13]. Exercising above AT increases the capacity of both lean subjects and individuals with obesity, to remove lactic acid from the circulation. Hyperlactatemia and lactic acidosis are among the precipitating factors of multi-organ failure, the most dreadful complication of COVID-19 [8]. Therefore, upregulating lactic acid disposal capacity constitutes a defense against multi-organ failure mainly for subject with a low AT as individuals with obesity [8]. Furthermore, in a diagram representing AT plotted against body mass index (BMI) (or percent of ideal weight), a linear increase can be observed in normal subjects, while a linear decrease can be documented in subjects with obesity [14], suggesting that in individuals with obesity the decrease in AT is proportional to the increase in body mass.

### Anaerobic threshold, lactic acid, respiratory exchange ratio (RER)

The intensity of physical work plays a role in the utilization of metabolic substrates. At low-intensity exercise (< 30% of VO_2_max), lipids deriving from adipose tissue store are used predominantly; at moderate-intensity exercise (40–65% VO_2_max) significant amounts of fats from both adipose tissue and intramuscular stores are used; at high-intensity exercise (> 70% VO_2_max), small amounts of fats are used, while glucose and glycogen become the predominant energy substrates [15].

AT indicates the VO_2_ level at which starts the anaerobic supplementation of the aerobic energy production. Both arterial lactate and lactate/pyruvate ratio increase for a given subject depending on his fitness and form of exercise [16]. It may be a useful index for evaluating daily life activity and in prescribing exercise regimen [17].

Methods using lactatemia and/or ventilation are reference methods to fix working intensities. They allow the detection of two lactic thresholds and two ventilator thresholds. The former threshold (lactic or ventilatory) is mainly useful for training programs in ill subjects, while the latter (lactic or ventilatory) is useful for training programs in healthy subjects and athletes [18]. Ventilatory threshold is used as an effort-independent physiological marker of the ability to perform submaximal, prolonged physical activity [19].

The aerobic energy system (below AT) produces the energy source adenosine triphosphate (ATP) by oxidative pathways, being configured for long-term steady work. The anaerobic alactic and lactic acid systems are less efficient than the aerobic system, producing quickly energy by nonoxidative pathways [20]. Their activation is short lasting, depending on the limited glycogen stores in human body and consequent to the accumulation of fatigue-related metabolites [e. g. H^+^ Pi and extracellular K^+^ [21]].

The most feared event complicating COVID-19 is multi-organ failure, which entails a failure of mitochondrial ATP production in many vital organs such as lungs but also heart, kidneys, gut and brain. COVID-19 in patients with obesity involves a higher mortality due to several factors among which the cellular respiratory chain failure predominates [22]. During multi-organ failure, lactic acid along with fatigue and stress-related metabolites (mainly H^+^) increases due to a switch toward anaerobic metabolism. To note that the switch takes place earlier in subjects with obesity (namely at lower workloads) and even at very low workloads (e.g., simply walking) in individuals with stage III obesity.

The Respiratory Exchange Ratio (RER) is the ratio between CO_2_ output and O_2_ uptake. Usually, it is considered during physical stress, providing information about AT and predominant fuel utilization (with the acknowledged limitation of not considering protein utilization). The classical criteria for detecting AT are the following:1) analysis of the straight-line relations of VCO_2_ vs VO_2_ (V-slope method) [23]; 2) inflection point on the minute ventilation (V_E_) vs oxygen consumption (VO_2_) diagram; 3) point of increase in end-tidal VO_2_ (PETO_2_); 4) point of increase in the ventilatory equivalent of O_2_ (V_E_/VO_2_) without a concomitant increase in the ventilatory equivalent of CO_2_ (V_E_/VCO_2_) [23].

Human muscles contain both slow twitch fibers (type 1) more fat burning, and fast twitch fibers (type 2) with a preference for carbohydrate. Lower exercise intensity recruits more type 1 fibers while highest intensities recruit an higher percentage of type 2 fibers, which are added to those of type 1 [20].

This review stems from exercise testing in subjects with obesity and emphasizes the relevance of AT as a drift point in the behavior of specific hormones and mediators. Building on that physiological evidence, it will summarize the research conducted up to date on the effects of physical training on metabolism and body composition of individuals with obesity at work outputs below and beyond AT. Finally, we will propose a combination of Aerobic Training along with Aerobic plus Anaerobic Training as appropriate exercise prescription in subjects with obesity undergoing rehabilitation.

The rationale of combining Aerobic and Anaerobic training (which entails “playing around the anaerobic threshold”) is particularly relevant in the COVID prevention and in the post-COVID rehabilitation of subjects with obesity. This is because training our muscles to dispose more quickly of lactic acid will increase the aerobic power of the mitochondria, and specific training programs tailored for individuals with obesity may improve their immunomodulatory capacity [7]. Moreover, sedentariness caused by lockdown periods has a deconditioning effect also in unaffected individuals, although it determines a striking burden in individuals with obesity or diabetes [24]. Recently, high-intensity interval training (HIIT) (brief bursts of vigorous intensity interspersed with periods of rest or low-intensity exercise) has been tested in subjects with obesity, showing positive effects on aerobic fitness and cardiovascular protection [25].

## Literature search

We searched the PubMed database for relevant literature. The search on obesity and AT was performed focusing in parallel on: 1) behavior of hormones and mediators during exercise testing; 2) effects of training at different intensities (that is below AT vs below and beyond AT); 3) effects of bouts of high-intensity exercise in relation to COVID-19.

We used the following keywords for the first search topic: (obesity AND adults AND exercise AND anaerobic threshold) AND plasma catecholamine OR epinephrine OR norepinephrine OR lactic acid OR potassium OR growth hormone OR GH OR leptin. Subsequently, a second search topic with the following keywords was performed: (obesity AND adults AND exercise AND anaerobic threshold AND physical training) AND growth hormone OR GH OR non-esterified fatty acids OR NEFA OR insulin resistance OR lactic acid OR leptin OR body composition. Finally, as last topic, a search using as keywords (Obesity AND COVID-19 AND High Intensity Exercise) was performed.

Only peer-reviewed articles written in English were included in the list of retrieved scientific studies. The final screening was based on the relevance of the identified items. Twelve articles were considered in the first search topic (McMurray RG et al. 2005, Connolly DAJ et al. 2012, Wasserman K 1984, Salvadori A et al. 1992, Salvadori A et al. 1991, Cahill BR et al. 1997, Salvadori A et al. 2003, Salvadori A et al. 2008, Hamilton MT et al. 2000, Salvadori et al. 2004, Felsing NE et al. 1992, Koppo K et al. 2010), ten articles were considered in the second search topic (Tamai M et al. 1993, Weltman A et al. 2008, Sideman L et al. 2002, Salvadori A et al. 2010, De Glisezinski I et al. 2003, Dengel DR et al. 1996, Salvadori et al. 2014, Salvadori A et al. 2015, Jelleyman C et al. 2015, Lanzi S et al. 2015) and five articles were considered in the third search topic (Rahmati-Ahmadabad S et al., 2020, Yang S, 2020, Wang M, 2020, Baena Morales S 2021, Kemmler W, 2021).

Considering these retrieved papers and the experience of our multidisciplinary team, we report on the following observations linking physical activity to anaerobic threshold in subjects with obesity, before and during COVID-19 pandemic, speculating on a potential protection from COVID-19 of above AT or of mixed aerobic and anaerobic training.

## Anaerobic threshold in subjects with obesity and normal subjects

During a progressive exercise testing, AT (former threshold) represents a “drift point” for many indexes (catecholamine, lactic acid, potassium and growth hormone).

### Plasma catecholamine pattern during exercise in obesity: its potential ominous role in COVID-19

Physical stress causes activation of the SNS. This elicits an increase in plasma catecholamine, which in turn contributes to increasing the heart rate, arterial pressure, and myocardial contractility [26]. In addition, due to SNS innervation of adipose tissue, physical stress promotes lipolysis and release of glycerol and free fatty acids (NEFA) into the circulation [27].

Exercise intensity is a key determinant of the catecholaminergic response. Power outputs exceeding the AT are associated with an exaggerated catecholamine activation [28] with an exponential rising of epinephrine (E) and norepinephrine (NE) concentrations [29]. Factors like degree of fitness, muscle conditioning, and degree of involvement of the muscular mass can influence catecholamine response [26–29].

In contrast to the lean subjects, individuals with obesity show a trend toward higher catecholamine response for lower work outputs before AT, while a lower catecholamine response for higher work outputs beyond AT [30].

During a progressive cycle-ergometer test in untrained young normal subjects, plasma epinephrine levels have been shown to increase by 180% from rest to AT, and by 950% from rest to maximal sustainable peak activity. In contrast, in subjects with obesity, the increments in epinephrine levels were 205% from rest to AT and 335% from rest to maximal sustainable peak activity.

Norepinephrine concentration increased, respectively, 215% and 550% in subjects with obesity while 162% and 920% in lean age-matched controls [30]. These data partly confirm those of Gustafson et al. [31] and may suggest that obesity can elicit different catecholamine responses depending on the intensity of the physical stress.

The altered catecholamine kinetics present in individuals with obesity at low workloads (with a more pronounced increment of both epinephrine and norepinephrine) may predispose to cardiac complications during COVID-19. In fact, firstly an altered catecholamine physiology was demonstrated during SARS-CoV2 infection [32]. Secondly, the high prevalence of Takotsubo syndrome in COVID-19 patients was documented. Takotsubo syndrome, which is often fatal, is characterized by ECG alterations (ST elevations, precordial T wave inversion), normal or near-normal troponin and high level of catecholamine. This syndrome may lead to acute cardiac failure with low cardiac output [33]. Thirdly, the psychological distress undermining our lives in COVID-19 era may increase the incidence of Takotsubo syndrome, mainly in individuals with obesity with a higher catecholamine response than lean subjects at low workloads [34]. In other words, individuals with obesity usually show higher catecholamine levels that lean counterparts during everyday life activities and this pattern may expose them to higher risk of cardiovascular complications during COVID, such as Takotsubo syndrome.

### Plasma lactic acid and plasma potassium during exercise in subjects with obesity: the potential role on prognosis of COVID-19

As stated above, plasma lactic acid increases when metabolism has shifted toward nonoxidative pathway, beyond the “lactate threshold,” to increase ATP production.

Lactic acid increases during a progressive exercise. In subjects with obesity, exercise beyond AT increases lactic acid production less than in controls at similar maximal power outputs [35]. This blunted response is due to the well-known insulin resistance, typical of subjects with obesity. Noticeably, increases in lactate anions play an important role in changing hydrogen ion concentrations in plasma and muscles [36] and, in turn, this could be relevant for the control of ventilatory function like ventilation during exercise. In the basal state, individuals with obesity produce more lactic acid than lean subjects do, mainly from chronically hypoxic adipocytes [37]. The high lactic acid outflow contributes to the development of blood acidosis and constitutes a predisposing factor to multi-organ failure in patients with obesity affected by COVID-19 [8].

Physical stress elicits a release of potassium ions (K^+^) from contracting muscles, which is proportional to the entity of work [38]. Potassium release depends on a mismatch between the potential charge of sarcolemma during muscle contraction and the ability of the Na^+^- K^+^ pump to keep pace with the rate of K^+^ loss and/or to a simultaneous acid–base change due to reduction in nondiffusible intracellular anions linked to phosphocreatine hydrolysis [39]. K^+^ reuptake by the Na^+^- K^+^ pump in contracting and noncontracting muscles, as well as kidneys, is able to counterbalance K^+^ release [40]. It is associated with the activity of α and β-adrenergic receptors, which, respectively, reduce and increase the Na^+^- K^+^ pump activity [40]. Through β-adrenergic stimulation, this catecholamine-driven K^+^ ions reuptake promotes and protects against hyperkalemia [40].

**Fig. 1:**
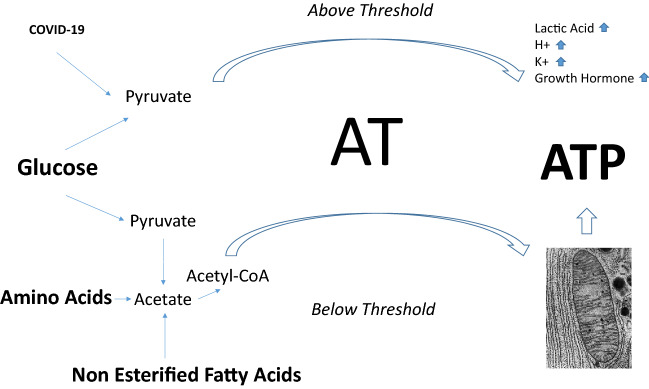
COVID-19 shifts metabolism toward anaerobic glycolysis eventually inducing metabolic acidosis and hypopotassemia. Bouts of physical exercise above AT cause a positive modulation of hormones and metabolites like, growth hormone, lactic acid, H^+^, K^+^

Static muscular contractions increase heart rate and arterial pressure [41] by means of a reflex initiated by the stimulation of Group III and IV afferents whose terminals are located in the interstitium of the working muscle [42]. The accumulation of K^+^ in the interstitium of the muscle is considered a possible important mechanism by which contractions activate Group III and IV afferents [43]. Both in normal weight subjects and individuals with obesity, a progressive physical exercise causes significant increases in plasma K^+^, although increases are lower in subjects with obesity [30] (Fig. 1).

Structural modifications in peripheral organs may contribute to the regulation of cardiovascular and respiratory parameters variables [44, 45]. In fact, quadriceps muscular biopsies in subjects with obesity are characterized by fiber hypertrophy and intracellular accumulation of neutral lipids [46–48]. Fiber hypertrophy has been associated with a higher density of the Na^+^- K^+^ pump sites [49]. This finding possibly explains the lower increase in plasma K^+^ in subjects with obesity seen during progressive exercise. Moreover, subjects with obesity show lower increases in plasma K^+^ at work outputs below AT with respect to controls, while higher increases than controls are seen in individuals with obesity beyond AT [30] (Fig. 1). This apparently divergent behavior could be explained by the greater muscular mass of subjects with obesity involved in the physical exercise. Higher muscle mass intensifies E and NE release at the lower outputs, and a lower increase in plasma K^+^ observed before AT could be due to enhanced muscular K^+^ reuptake by *β* receptors. Beyond AT, the lower increase in plasma E and NE is coupled with a greater increase in plasma K^+^. The opposite seems to occur in controls [30].

The altered K^+^ kinetics of individuals with obesity may concur in worsening the hypopotassemia present in COVID-19 [50]. In fact, the occupancy of ACE2 receptors by SARS-CoV2 binding via the viral S protein causes an imbalance in the Renin-Angiotensin-System determining a prevalence of ACE1 (which increases RAS activity) [50]. The increase in RAS activity leads to an increase in aldosterone and consequent increase in renal excretion of Na^+^, water and K^+^, ending up in hypopotassemia, which may last much longer after remission of COVID-19 [51]. An imbalance of the RAS system may also cause a pro-thrombotic state contributing to the multi-organ failure based on metabolic acidosis [52].

Plasma K^+^ is also involved in the incremental ventilatory response during physical activity [53, 54]. In subjects with obesity undergoing a progressive incremental exercise, the ventilatory response is less robust than in controls. The difference is maximal when ventilation has adjusted for the fat-free mass [35].

To summarize the role of lactate and K^+^, they are involved in the regulation of V_E_ with a peculiar pattern linked to the morphology of muscle fibers (hypertrophy) and insulin resistance (basal increase in lactate and reduced increase in lactic acid at maximal work outputs) [35, 53, 54]. The alterations of both K^+^ and potassium kinetics may play specific roles to worsen the prognosis of COVID-19 in subjects with obesity.

### Growth hormone (GH) during exercise in subjects with obesity: its potential role in COVID-19 prognosis

GH is one of the key regulators of body fat and lean tissue. A variety of conditions affect GH secretion like, gender, pubertal stage, age, sleep, nutritional status, body composition, body temperature, fitness, gonadal steroids, insulin and IGF-1 [55–57].

Physical activity stimulates GH secretion [58]. The magnitude of GH response to exercise depends on its duration and intensity, as well gender, fitness and age [59]. An open question regards the hypothesis that GH response implies a threshold of exercise intensity, or a linearly increase along with increasing intensity of work [60–62].

Obesity blunts the GH response to exercise [55]. Similarly, abdominal visceral fat has been identified as a responsible for the reduction both in spontaneous and stimulated GH secretion [55, 56]. In lean subjects, during a progressive exercise, a significant GH increase is detectable from rest to AT, and a further significant increase vs rest and vs AT up to maximal activity. In subjects with obesity no significant GH increase occurs from rest to AT, while a slight but significant GH increase is seen from AT to peak activity when expressed as area under the curve (AUCs). Compared to lean controls, GH response to exercise is null before AT, and significantly lower beyond AT in subjects with obesity [63].

The blunted GH response may partially explain the greater severity of COVID-19 illness of individuals with obesity, mainly if they are elderly and of male sex [64]. The proposed underlying mechanism is a defective immunomodulatory effect in GH deficient individuals [64]. In fact, growth hormone plays a fundamental role in immune modulation. Therefore, exercising above the AT may increase GH response in subjects with obesity, and it is conceivable that the higher GH response may induce a positive immunomodulatory effect [64].

### Leptin during exercise in subjects with obesity: relationship with COVID-19

Leptin is a hormone involved in the regulation of body weight and satiety [65], mainly secreted by adipocytes [66, 67]. It decreases when energy intake is restricted, and increases as body fat accumulates [67]. It is well known that obesity is associated with an increase in circulatory leptin levels and prompts a condition of leptin resistance [67]. Both acute physical exercise and prolonged physical training promote a decrease in plasma leptin [68–70]. Growth hormone is a factor able to contrast the extent of leptin reduction due to physical stress [71], as well as the increase in intracellular products from glucose metabolism from bouts of work beyond AT, in absence of improvement in insulin sensitivity for glucose [72].

The higher leptin levels present in individuals with obesity may play a role in the severity of COVID-19 [73]. In fact, besides regulating appetite and metabolism, leptin also signals via the Jak/STAT and Akt pathways and modulates T cell function. Recent studies demonstrated that leptin upregulates expression of inflammatory cytokines in monocytes, and potentially contributes to ensuing the so-called cytokine storm, which often preludes to a negative outcome of COVID-19 [74].

## Fat_max_ and high-intensity interval training (HIIT) in subjects with obesity: its potential role in COVID-19 prevention

Herein, we have considered AT as a corner stone because it implicates modifications in hormones and mediators responses during a progressive exercise. Some authors have focused on exercise modalities utilizing other training models, like continuous work at Fat_max_ (exercise training at intensity eliciting maximal fat oxidation) and high-intensity interval training (HIIT).

Fat_max_ exercise training is performed at 60–70% of maximal heart rate. This, particularly in subjects with obesity, could be an exercise threshold already across, if not clearly beyond, the achievement of AT [75].

About HIIT, repeated bouts of maximal exercise have shown a decreasing activity of glycolysis, with a shift toward oxidative phosphorylation [76]. Thus, HIIT is already considered as a modality of aerobic training [76].

In subjects with obesity, adaptation to moderate and high-intensity interval exercise can differ interindividually and high-intensity exercise training sessions are not feasible for all patients with obesity undergoing a training program, especially in subjects with class III obesity [77].

We previously showed that in subjects with second and third class of obesity, eight cycling sessions (spread over 2 weeks) of a moderate-intensity continuous training were both effective in improving aerobic fitness and fat oxidation rates during exercise [78]. In this study, HIIT had tendency toward promoting a more marked increase in VO_2max_ compared to Fat_max_ training (+ 8% and + 4%, respectively). This improvement is likely related to exercise intensity [79] and highlights the following: 1) HIIT is a feasible and time-efficient training in class II and III subjects with obesity, as previously shown in overweight and class I subjects with obesity [80]; 2) in those obesity categories, HIIT improves aerobic fitness [81–83]; 3) promoting HIIT early after initiation of training programs can help to reverse the low aerobic fitness in individuals with obesity [84]. While HIIT seems preferable at Fat_max_ when compared to moderate-intensity training, only the latter induced a significant reduction in fasting insulin and insulin resistance [85], suggesting the importance of exercise duration for improving insulin sensitivity in subjects with obesity. The insulin-sensitizing effect of Fat_max_ could be related to decreased levels of plasma non-esterified acids (NEFA) [86, 87]. Finally, considering the need of increasing training variety and adherence in the real world setting [88], HIIT and moderate-intensity continuous training may be two complementary training tools.

In relation to COVID-19, both Fat_max_ and HIIT seem appropriate trainings modalities in subjects with obesity. The former, with a work intensity at 60–70% of maximal heart rate reach the AT in individuals with obesity, and all the benefits illustrated in the previous chapter. The latter (HIIT) being a modality of aerobic training reduces the leptin level in subjects with obesity, ameliorating the negative effect of leptin on immune modulation [73, 74]. In particular, in a social condition of lockdown and distancing with gyms and sites for aggregation unavailable, HIIT, namely a training model requiring less time to obtain the metabolic and immunomodulatory effects, seems the most appropriate way of exercising.

## Playing around the anaerobic threshold: a tool against Sars-cov2 infection and complications

Regular physical exercise associated with diet is fundamental strategy in the management and rehabilitation of subjects with obesity. The American College of Sport Medicine recommends the addition of resistance exercise to a regular program of aerobic training [89]. Mild, aerobic exercise increases lipolysis by means of increased plasma catecholamine and lowers plasma concentrations of insulin an antilipolytic hormone, while plasma NEFA concentrations drop after exercise [90–93].

To our knowledge, only few studies analyzed the effects of low- or high-intensity training in individuals with obesity. To investigate the effects of moderate-intensity training below and beyond AT on body weight, body composition and metabolism in adult subjects with obesity, this protocol has been used to better clarify the isolate impact of physical activity on weight loss and biochemical modifications at different work outputs. It could be also adopted for the rehabilitation of post-COVID-19 patients with obesity, based on all considerations previously discussed.

Two experimental training conditions have been tested: (a) moderate-intensity training below AT and (b) moderate-intensity training with a single bout of high-intensity exercise, beyond AT. The study protocol was conducted during a 4-week hospital stage (two cycle-ergometer sessions/day of 1/2 h, for 6 days/week) with one group training at constant work-output attaining 70% AT heart rate, while the other underwent 25 min training at 70% AT and a final exercise bout of 5 min at 85% of maximal heart rate. Noticeably, there was no significant difference in the total amount of performed work when referred to fat-free mass between group (a) and group (b) after the period of training [94].

After the 4-wk reconditioning program, both types of exercise determined an increase in AT, of the maximal peak of activity, of the VO_2_ max, associated with a decrease in body weight. In particular, the decrease in fat mass was significantly higher following moderate-intensity training plus single bout of high-intensity exercise (b) compared to exercise performed only at moderate intensity (a) [93].

## 5.1 Growth hormone (GH) after training in individuals with obesity

As reported before, subjects with obesity achieve a null GH response exercising below AT, and a clearly lower response when they exercise beyond AT when compared to lean subjects. This confirms that AT appears as a point-break for the modification in the GH response to exercise in subjects with obesity [63].

**Fig. 2:**
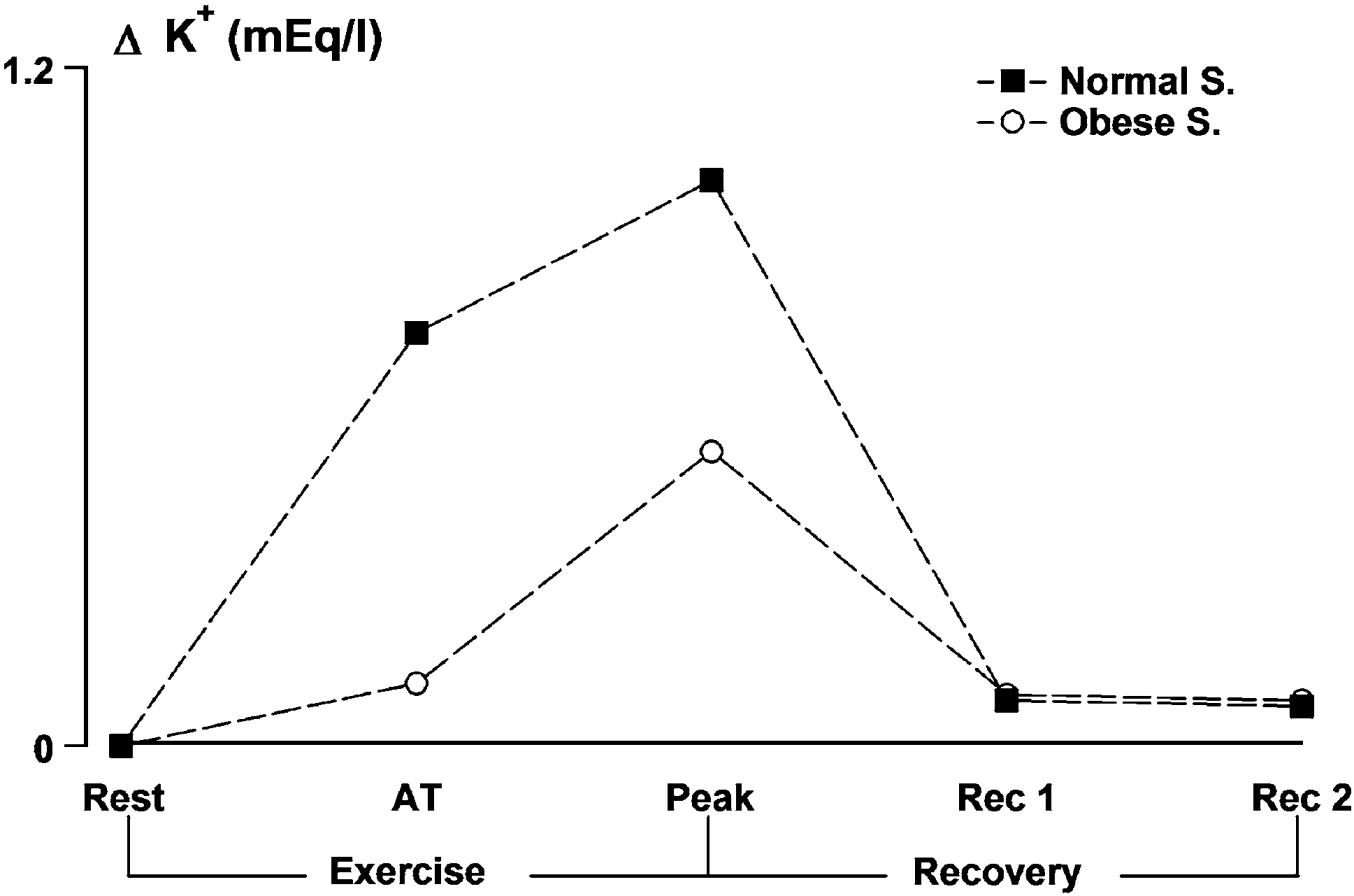
K^+^ modification during exercise testing before and after anaerobic threshold

After four weeks of training, the GH response to exercise of moderate-intensity training plus single bout of high-intensity exercise (b) was higher than GH response following only moderate intensity (a) in individuals with obesity. In fact, adding single bouts of strenuous work at the end of each regular session of moderate intensity is able to evoke a significantly higher GH secretion in response to exercise intensity exceeding AT as shown in Fig. 2 [63]. The restoration of GH secretion will improve the immunomodulatory capacity in individuals with obesity.

### Non-esterified fatty acids (NEFA), insulin resistance, lactic acid after training in subjects with obesity.

**Fig. 3:**
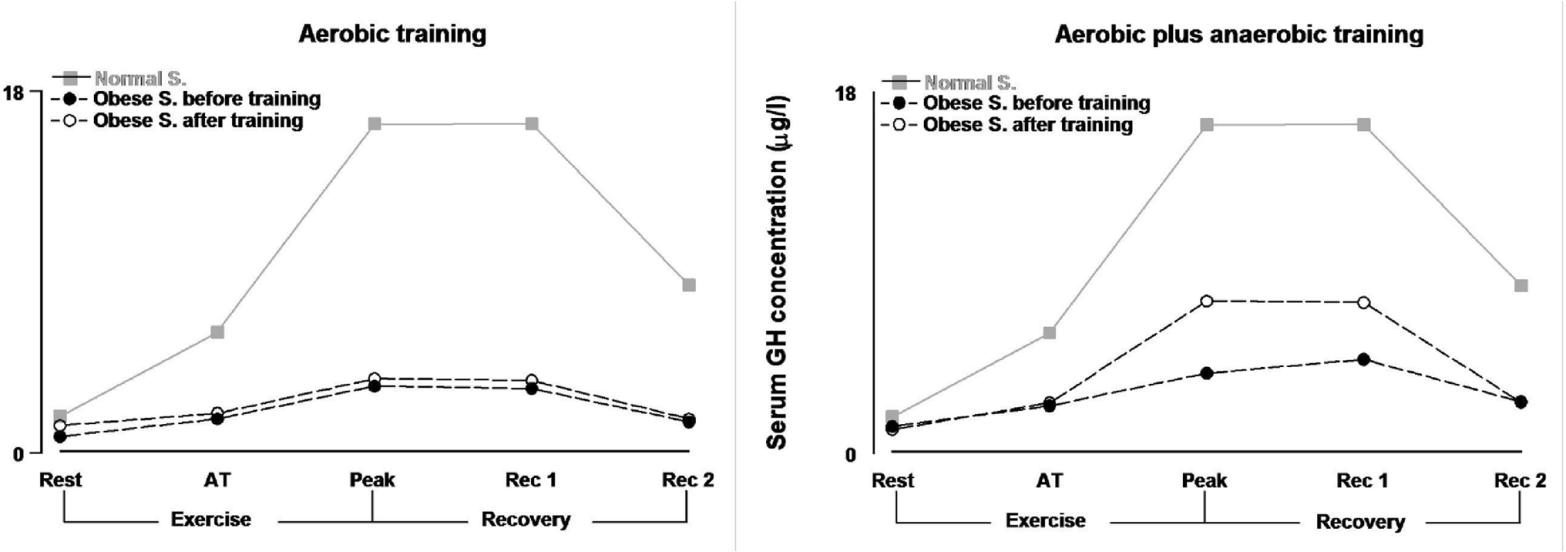
Serum GH concentration during exercise testing before and after aerobic training (left panel), before and after aerobic plus anaerobic training (right panel) in subjects with obesity vs normal subjects

Aerobic training reduces circulating NEFA [90–93] both in resting condition and during physical activity (Fig. 3). An increasing amount of lipids oxidized during exercise may cause a negative balance between a slow mobilization of fatty acids from adipose tissue and their rapidly increased extraction by the skeletal muscle in activity [95]. Moreover, aerobic training reduces insulin resistance (HOMA 2-B) and increases lactic acid during exercise beyond AT [93]. In accordance with other studies [96, 97], these results suggest that both mitochondrial oxidative capacity and glucose utilization have improved following moderate aerobic exercise.

In contrast to moderate-intensity training (a), a period of moderate training with brief periods of work beyond AT (b) seems to promote different modifications, like an increase in circulating NEFA (Fig. 3), a significantly higher fat mass loss, no change in HOMA 2-B, a total lower level in lactic acid during exercise [93]. Lactate is a major energy source, and its lower levels during exercise after aerobic and anaerobic training may even be due to a higher clearance, as observed in trained athletes [98]. Training the subjects with obesity to better dispose lactic acid and potassium is a protective mechanism in case of SARS-CoV2 infection [7, 8, 30].

Overall, a moderate-intensity training constantly below AT (a) seems to promote an improvement in NEFA utilization and, at the same time, an improvement in insulin sensitivity, with a poor fat mass reduction [93]. A moderate-intensity training with bouts of high intensity (b) (that is beyond AT) seems to yield no clear improvement in metabolic profile, while fat mass reduction becomes more efficient [93]. It is conceivable that the physical stress due to physical exercise beyond AT does not improve insulin sensitivity, while the increased spillover of lipolytic mediators like catecholamines and GH promotes a lipid mobilization which exceeds their dynamic utilization [93].

### 5.3 Leptin after training in subjects with obesity

After a period of training, serum leptin decreases in obesity together with fat mass. Aerobic exercise (a) promotes a significant decrease in leptin and a slight fat mass loss, while aerobic and anaerobic activity and (b) promotes a slight reduction in leptin with a higher fat mass loss [94].

In the absence of dietary restrictions, physical training decreases leptin hypersecretion and fat mass. Leptin decrease seems to be related only to the intensity of performed work, but dissociated from fat mass loss after training, although the relationship between physical exercise, leptin and fat mass is very complex [99, 100].

The robust reduction in leptin after moderate exercise training alone (a) linearly correlates with the total amount of performed work. The higher the amount of work, the higher the reduction in leptin [94].

The slight reduction of leptin after moderate plus high-intensity training (b) implies an opposite trend: the higher the amount of work, the lower the leptin reduction [94].

This consideration may agree with the notion that factors like GH as well as an increase in intracellular products of glucose metabolism (e.g., high lactic acid due to work beyond AT) contrast the reduction in leptin caused by physical stress.

## Can physical exercise around the anaerobic threshold prevent COVID-19 infection and serious?

**Fig. 4:**
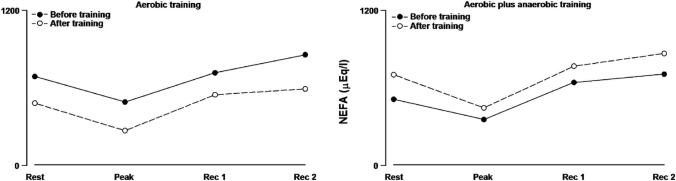
Plasma NEFA concentration during exercise testing before and after aerobic training (left panel), before and after aerobic plus anaerobic training (right panel) in subjects with obesity

Since AT is a point-break leading to the release of metabolites and hormones affecting the immunomodulatory capacity of individuals with obesity, it is mandatory to exceed the AT level performing specific training programs. We speculate that adding short bouts of exercise above AT to medium-intensity continuous exercise below AT (50% of VO_2_ max) may permanently reduce lactic acid and H^+^ [93] and increase growth hormone secretion (Fig. 4) [63]. Those hormone and metabolite changes will improve the host defenses against viral infections and reduce the likelihood of metabolic acidosis following SARS-CoV-2 contagion. In contrast, the impact of a lower reduction of leptin levels on the immunomodulatory capacity of individuals with obesity, obtained adding bouts of exercise above the AT, is hard to explain at present.

## Physical exercise and recovery from cardiopulmonary sequelae of COVID -19 in patients with obesity

Based on the above considerations, the opportunity of training programs including bouts of exercises above the AT applies also after a COVID-19 illness in individuals with obesity. In fact, firstly, cardiopulmonary sequelae are frequent in the post-COVID period, and frail individuals, like subjects with obesity, necessitate of muscular and cardiopulmonary rehabilitation. Secondly, a prolongation of symptoms like asthenia, muscle aches, intermittent low-grade fever, shortness of breath and cognitive dysfunction may persist up to 4–6 months after the negativization of SARS-CoV2 throat swab, identifying the newly called “Long-COVID” syndrome [101]. Thirdly, the possibility of re-infection with a new viral variant [102]. All those factors make useful the implementation of physical exercise programs both to prevent re-infection and to rehabilitate post- COVID individuals with obesity.

## Conclusions

In all vertebrates, the start of any movement is an anaerobic process, being the anaerobic energy system the source of emergency-response energy. During evolution of species, the anaerobic power and capacity of vertebrate’s genera has been an essential component of survival [103] pinpointing on the importance of the anaerobic threshold regulation.

However, current understanding of role of AT is generally limited as the knowledge of the underlying complex interplay of biochemical reactions and responses [104]. Nevertheless, in physical rehabilitation AT can be easily monitored via heart rate and its changes during training and testing and provides relevant information on relative and absolute intensity of work, including efficiency and safety [104]. Herein, we showed that AT can be considered not only a switch for respiratory gases and for lactic acid, but, presumably, it also represents a trigger for the activity of various hormones and biochemical mediators. The occurrence of COVID-19 pandemic prompted the present work, and the suggestion of adding bouts of exercise above AT is strictly enforced in subjects with obesity both to prevent SARS-CoV2 infection and to combat COVID-19 sequelae and re-infection during rehabilitation periods.

In accordance with recent reports, scientific evidence emphasizes the effectiveness of physical training in the treatment of obesity, particularly during global pandemics [105]. This review reports on the differential response of lean subjects and individuals with obesity during different modalities and intensities of physical exercise as the pattern and modifications of mediators, hormones and metabolites are concerned. We aimed to describe the different biologic responses to physical stress when exercising “up and down” the anaerobic threshold and to suggest a prescription of physical activity tailored for rehabilitation of individuals with obesity.

The distillation of our review in a practical suggestion entails the opportunity to prescribe a period of moderate (below AT) plus high-intensity (beyond AT) training as first approach in subjects with obesity without major comorbidities. The rationale behind this approach is to search for a maximal stimulation of lipolysis. Subsequently, it is conceivable to stimulate metabolic rate with a prolonged moderate-intensity training alone, to further improve fat loss and ameliorate insulin and leptin resistance. This approach would be particularly valuable for individuals with obesity during the COVID-19 pandemic as concerns both prevention and follow-up. Interestingly, the considerations concerning COVID-19 might be extended to other viral pandemics, e.g., the influenza virus’s pandemics [8]. This is because adding bouts of high-intensity exercise (above AT) will induce positive immunomodulatory changes.

Several recent reports are along the line of the proposed training program for the prevention of COVID-19 [106]. In particular, older individuals may benefit of appropriate HIIT programs during COVID-19 pandemic [107]. In a multinational study, Wang et al. propose HIIT along with moderate aerobic training (Fat_max_) as an approach that reduces infection rates, underlying pathologies and chance of mortality associated with COVID-19 [108], while Baena-Morales identifies several training modalities for different target groups [109]. Finally, Yang et al. clearly showed the dramatic impact on body weight of forced sedentariness during lockdown periods [110].

Clearly, there are also some limitations and contraindications to the suggested training program: firstly, individuals with obesity may have several comorbidities like hypertension or ischemic heart disease; secondly, subjects with grade II and III obesity may have biomechanical impediments to perform high-intensity physical activity; thirdly, the psychological reluctance of subjects with obesity to perform physical activity.

In conclusion, moderate- and high-intensity exercise training may be two complementary tools, to optimize the management of individuals with obesity, in the outpatient or in the rehabilitation ward settings, particularly useful during COVID-19 pandemic.
